# Inexperienced water users can “Float to Live” in realistic open water conditions

**DOI:** 10.1186/s12889-024-19409-6

**Published:** 2024-07-29

**Authors:** Clare Eglin, Heather Massey, Geoff Long, Adrian Mayhew, Michael Tipton

**Affiliations:** 1 Extreme Environments Laboratory, University of Portsmouth, Portsmouth, UK; 2 Surf Life Saving GB, Exeter, UK

**Keywords:** Drowning prevention, Water safety, Floating, Water immersion

## Abstract

**Background:**

The RNLI “Float to Live” campaign is based on research conducted in indoor pools with experienced open water swimmers. Study 1 investigated whether the RNLI “Float to Live” guidance would enable less experienced individuals to float in realistic open water conditions. Study 2 examined the separate effects of practice and coaching on floating competence.

**Methods:**

Study 1: Inexperienced water users conducted floats in either still, open fresh (*n* = 22) or open sea water (*n* = 13), followed by moving sea (*n* = 6) or fresh water (*n* = 5). Participants undertook three 2-min floats in still water wearing swimwear and one clothed float: 1) naïve; 2) following RNLI “Float to live” messaging; 3) individual float coaching; 4) simulated fall wearing summer clothing. In moving sea water, participants undertook two floats equivalent to Floats 3 and 4. In moving fresh water, participants undertook 3 floats: 1) naïve; 2) following “defensive floating” coaching; 3) simulated fall wearing summer clothing. Study 2: Two groups matched for skinfold thickness undertook three 2-min floats in a flume wearing swimwear. PRAC group (*n* = 12): 1) naïve; 2) following float practice; 3) float coaching; COACH group (*n* = 11) coaching followed by practice. Floating difficulty, confidence, competence, “efficiency” and perceived exertion were analysed using either a Friedman test or mixed model ANOVA.

**Results:**

In both fresh water and sea water, participants’ floating competence and confidence increased after viewing the RNLI messaging, it was further improved with individualised float coaching. The additional helpful instructions included: 1) “head back with ears submerged”; 2) “relax”; 3) “breathe normally”; 4) “it is OK if your legs sink”; 5) an accurate description of sculling for “active” floaters that needed it; 6) spread arms and legs for stability. The simulated fall with clothing did not impair floating competence. No difference in floating competence was seen between PRAC and COACH, though confidence may be increased sooner in COACH.

**Conclusions:**

The RNLI float advice can be applied in realistic open water settings by less experienced water users. Additional content could be included to make the messaging even more effective.

**Supplementary Information:**

The online version contains supplementary material available at 10.1186/s12889-024-19409-6.

## Introduction

There are an estimated 236,000 annual drowning deaths worldwide [26], with 226 accidental drowning fatalities occurring in the United Kingdom in 2022 [15]. Approximately half of the UK drowning victims had not intended to go into the water and were therefore not prepared [14]. One of the potential causes of drowning is the initial cardio-respiratory responses to cold water immersion, otherwise known as the cold shock response (CSR; [19],). The CSR comprises an inspiratory gasp (of approximately 2.5 L), followed by uncontrollable hyperventilation during which voluntary breath holding is severely compromised thus increasing the risk of drowning [19]. Heart rate, blood pressure and workload of the heart are also increased and arrhythmias may occur which may precipitate a cardiac arrest in individuals with underlying cardiovascular disease [19]. Whilst the responses peak in water temperature between 15 °C and 10 °C [20], the CSR can occur in warmer water [11] and therefore most of the open water around the UK is cold enough to evoke the CSR, even in summer.

The risk of drowning is increased if individuals attempt to swim when the CSR is at its peak, waiting 90 s for the hyperventilation to subside improves subsequent swimming ability [7, 20]. To prevent drowning, the Royal National Lifeboat Institution (RNLI) has, based on the research noted above, been promoting a “Float to Live” message since 2017. This aims to keep the airway clear of the water on initial immersion by encouraging floating [3] until the CSR has subsided, and before trying to swim [7]. The RNLI “Float to Live” messaging provides instruction about how to stay afloat – undertaking the minimum amount of activity possible, thereby minimising the challenge to the airway and strain placed on the heart, as well as maximising the amount of air retained within clothing layers, which provides additional buoyancy [2]. However, this advice is based on research conducted in indoor pools with confident and experienced open water swimmers. The current study tested the hypotheses that the RNLI “Float to Live” messaging would enable inexperienced water users to float in realistic open water conditions (still and moving open fresh water and sea water), and that floating confidence and competence could be further improved with individual float coaching. Since this involved an inevitable component of practice, in a second study, the effectiveness of float practice and coaching on floating confidence and competence were compared. It was hypothesised that coaching would improve floating competence and confidence more than practice.

## Methods

Two separate studies were undertaken. In Study 1, a repeated measures experimental design was employed whereby participants undertook four sequential floats in open water. The effect of the RNLI “Float to Live” messaging on floating competence, confidence and difficulty was assessed compared to a naïve float. To determine whether floating could be further improved, participants were given individual float coaching and floating assessed again. Feedback on the instructions that helped participants float was collated to improve the “Float to Live” messaging. A final float was then conducted to determine whether the combination of instructions and practice would enable participants to float in a more realistic scenario after a simulated fall. A subset of participants also undertook floats in moving sea/fresh water. Logistic constraints meant that it was not possible to include a control group in the open water testing, to address this a second study was conducted. In Study 2, the separate effects of practice and coaching on floating competence, confidence and difficulty were investigated using a between group repeated measures, counter-balanced, experimental design. The results from Study 1 indicated that floating was improved the after coaching compared to the RNLI messaging and that body fat influenced floating technique. Therefore, two groups matched for skinfold thickness undertook three floats in a swimming flume, both groups undertook a naïve float and were then given time to practice (PRAC) or given coaching (COACH) prior to their second float. PRAC were then given coaching and COACH allowed to practice prior to their third and final float.

### Participants

In Study 1, a total of 25 participants undertook four floats in still open water (fresh or sea water). Six of these participants undertook a further two floats in moving sea water and five participants undertook a further three floats in moving fresh water. The details of the floats conducted in moving water are given in the supplementary material. In Study 2, 23 participants undertook three floats in an indoor swimming flume.

Volunteers (men and women aged 18 to 60 years) were recruited from University staff and students and the general population, they provided informed written consent prior to their participation. A favourable opinion was given by the Faculty of Science and Health, University of Portsmouth Research Ethics Committee for both studies (SHFEC-2022–066 and SHFEC-2023–005).

The minimum water experience for participants undertaking floats in still water (open water or flume) was to be comfortable in chest-deep, still water. Participants’ maximum water experience was recreational swimming with no regular swimming in open water (other than the odd dip on holiday) and no competitive swimming (indoor, outdoor or triathlon/duathlon). Volunteers were also excluded if they had any cardiovascular or pulmonary disease, asthma triggered by cold or exercise, family history of sudden cardiac death, musculo-skeletal injuries that may impair movement in water, or had a fear of water.

Participant demographics (age, sex, self-reported race) were recorded and anthropometric measurements were made in a private room and included measurements of height (SECA 213, SECA, UK), body mass (SECA 899, SECA, UK), circumferences of the waist, hip, chest, thigh and arm (anthropometric tape, Rosscraft, Canada) and skinfold measures taken using callipers (Harpenden skin fold calliper, Baty International, UK) at the biceps, triceps, subscapula, supra iliac and thigh. All anthropometric variables were measured and recorded by the same researcher for each study according to the guidelines produced by the International Society for the Advancement of Kinanthropometry.

### Study 1

#### Still open water floats

Twenty-two participants conducted floats in open still fresh water (a lake or lagoon), and thirteen participants conducted floats in sea water in Langstone harbour (during slack water) between July and September 2022. All floats were on the back, face up. Participants undertook four floats in still water, each lasting 2 min. The first three floats (standard floats) were undertaken wearing swimwear and neoprene shoes with neutral buoyancy, and the final float wearing summer clothing (T-shirt and shorts, with at least the T-shirt being dry). Prior to each float, participants were instructed to: “stay at the surface with the minimum effort that you need to keep your head above water for 2 min”. They then walked into the water until they were waist deep and were then given a countdown to start their float. The first float was conducted in waist to chest deep water, if they floated in a vertical position, participants were moved to deeper water (but only if they were comfortable to do so). For the final float (simulated fall), participants stepped into deep water from a platform (stairs or mega stand up paddleboard [SUP]) with a freeboard of no more than 10 cm, and were given the additional instruction to guard their mouth and nose with their hand as they entered the water to prevent a glossopharyngeal reflex. For safety reasons, the simulated fall was conducted last and only if the participant, researchers and experienced swim teachers were happy for them to do so. Participants who were not confident to step into deep water undertook a standard float wearing clothing. The floats were conducted in the order shown below so that the instructions given were incremental and, for safety reasons, the simulated fall occurred after a combination of instruction and practice:

1. No instruction (naïve)
2. After viewing the RNLI “Float to Live” video and poster
3. Following coaching from an experienced swim teacher and qualified open water swim coach
4. Simulating a fall wearing summer clothing (shorts and T-shirt)

Prior to their second float, participants watched the RNLI’s “Float to Live” video [17] and looked at the associated poster. They were allowed to watch the video as many times as they wished and spend as long as they wanted looking at the poster. Before attempting the third float, coaching was provided by an experienced swim teacher, qualified open water swim coach. This was individualised to help the participant improve their floating technique. Most of the instruction was given out of the water and included showing them short video clips of their previous floating attempts to aid their learning. Some participants were given extra in-water instruction. Between their floats, participants dried off, put on a robe and were offered hot drinks to warm them up. Participants did not undertake their next float until they felt warm and therefore the duration between floats varied between individuals and with weather conditions.

Ten participants volunteered for still water floats in both fresh water and sea water and for logistic reasons they undertook their floats in fresh water first (mean 17 days [range 2 to 41] prior to their sea water floats). Since they already had float coaching (from the RNLI video and swim teachers), they only completed two floats in sea water, one standard float and one wearing clothing after a simulated fall (equivalent to Floats 3 and 4 above).

**Fig. 1:**
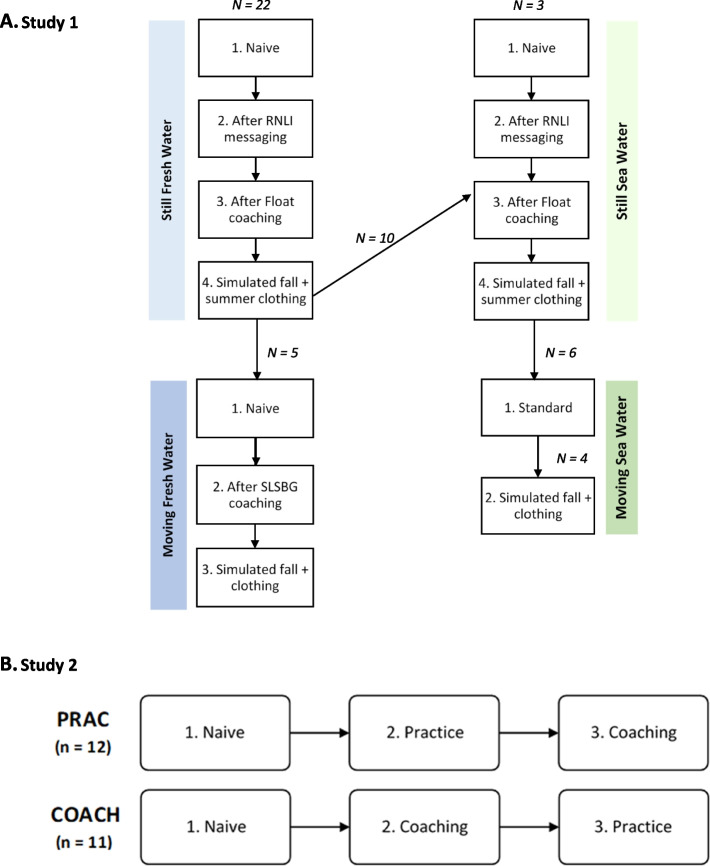
Order of floats undertaken by participants in Study 1 (**A**) for each of the conditions (still and moving fresh water and sea water). Order of floats undertaken by participants in the PRAC and COACH groups in Study 2 (**B**). The number of participants undertaking each float is also shown. In Study 1, one participant undertook floats in all conditions (still and moving fresh and sea water). Note: the methods and results for the floats in moving water are given in the supplementary material

A subset of participants also undertook floats in moving open water after their floats in still water. The methods and results for the two additional floats in moving sea water (*n* = 6) and three additional floats in moving fresh water (*n* = 5) are given in the supplementary material. The order of floats undertaken by the participants for each of the conditions is shown in Fig. 1A.

### Study 2

Twenty-three volunteers undertook three test floats wearing swimwear in a swimming flume (SwimEx 600-T Therapy Pool, length 4.2 m, width 2.3 m and depth 1.5 m) containing fresh water at 31 °C with no flow between March and May 2023. Participants attended an initial session in the laboratory where anthropometric measurements (as detailed above) were taken and subsequently used to create matched pairs of volunteers (matched for sum of skinfolds). One of the matched pairs was then randomly assigned (by the flip of a coin) to the practice session first (PRAC) or the coaching session first (COACH).

Participants entered the water via a ladder and stood in the water (between waist and chest depth) before commencing their float. The first float was naïve with no prior coaching or practice allowed. Following this they either practiced their floating technique with no coaching for up to 5 min (PRAC) or were given individualised float coaching by a swim teacher (COACH) for up to 5 min before undertaking their second 2 min float. Participants in the PRAC group were then given individualised float coaching, and those in the COACH group practiced their floating with no additional instruction (both for up to 5 min) before undertaking their final float. Each float lasted for up to 2 min and Floats 2 and 3 did not commence until heart rate had returned to baseline levels. The order of floats undertaken by both groups are shown in Fig. 1B.

### Measurements

Air temperature was measured close to the site of water entry using a wet bulb globe thermometer and recorded every minute on a data logger (Squirrel, Grants Instruments, UK). At Langstone harbour, wind speed and direction were measured at an adjacent building [24] and recorded prior to each float. The fresh open water sites were relatively sheltered therefore wind speed was not measured.

Water temperature was measured using calibrated thermometers at depths of 5 cm and 50 cm placed adjacent to the participant and recorded for each float. Specific gravity of the water was measured on each testing day using a refractometer. Water flow was measured at the point of water entry using a flowmeter (Model 001 Flowmeter, Valeport, UK). In the sea, wave height adjacent to the participant was assessed over a 20 s period during the float (retrospectively from video analysis) using a graduated pole, or estimated from the height of the SUP freeboard.

Prior to any floats or instruction, participants completed a pre-float questionnaire asking them to indicate their current participation in water-based activities*.* Self-reported water confidence in waist deep water and in water out of their depth using a 5-point Likert scale (1 = very unconfident; 2 = unconfident; 3 = neutral; 4 = confident; 5 = very confident). Participants were asked whether they could swim 25 m in a heated swimming pool without putting their feet down, and also to indicate their swimming ability on a scale of 1—10, where 1 was “I cannot swim at all” and 10 was “I am a great swimmer”. Participants were asked how difficult they find floating which was reported using a 5-point Likert scale (1 = very easy; 2 = easy; 3 = neutral; 4 = difficult; 5 = very difficult), and how much confidence they have in their ability to float, again using a 5-point Likert scale (1 = very unconfident; 2 = unconfident; 3 = neutral; 4 = confident; 5 = very confident). Finally, they were asked to list the actions they thought they needed to take in order to float. In Study 2, participants were also asked if they had tried to float before and if so, how long ago that was. Prior to each float, participants were asked to rate how difficult they thought their next float would be and how much confidence they had in their ability to float.

All floats were filmed using two mobile cameras (GoPro HERO8, GoPro USA) which were positioned to obtain an aerial and a close up or underwater view of the participant. Floating competence was assessed over the last 30 s of each float by the same two researchers (CE and HM) from analysis of the videos using a slightly modified version of the Moran floating scale (for score 1–2 the word excessive was replaced with energetic as this was a more accurate description of the activity; [12]). During some of the floats in Study 2, the movement of the participant set up a standing wave in the flume which interfered with floating. In these cases, if there had been no change in float technique, floating competence was assessed earlier when the water was calmer.

Immediately after their float, participants were asked to rate their perceived exertion (RPE) using the 0–10 Borg [4] scale, floating difficulty, whether they thought the float was more or less difficult than they anticipated (1 = a lot less difficult; 2 = less difficult; 3 = about the same; 4 = more difficult; 5 = a lot more difficult) and floating confidence. They were also asked which instructions or practice helped them to float (except after the first float when no instruction was given) or if any other information (not given) would have been useful. In Study 2, after the third float, participants were also asked whether practice or instruction helped them float the most and in which order they would be most beneficial.

Floating “efficiency” was estimated from floating competency divided by RPE for each float. Where the Moran score or RPE was 0, this was entered as 0.1 so that a value for floating “efficiency” could be obtained.

In Study 2, heart rate was measured using a Polar heat rate monitor (Polar H10, Polar, UK) and recorded at 30 s intervals during the three floats. Floating angle of the torso (from shoulder to hip) viewed from a window at the side of the flume was estimated in the final minute of each float using a protractor.

The float coaching given to the participants by the swim instructors was recorded, transcribed and coded into different themes. These corresponded to instructions regarding head position, relaxing, breathing, limb movement, leg position, arm position and core activity. These were then compared to the instructions reported by the participants as useful, in order to identify key phrases which enabled participants to improve their floating competence.

#### Data analyses

Pre-float floating difficulty and confidence, floating competence (Moran scale), RPE, post-float floating difficulty and confidence were compared between floats within conditions using a Friedman test (with post hoc Wilcoxon tests if appropriate) when three or more floats were compared, or Wilcoxon tests if only two floats were compared. Comparisons were also made between the final two floats in fresh water and the two floats in sea water. In Study 2, between group comparisons were made using a mixed model ANOVA with pairwise comparisons. Sphericity was assessed using Mauchly’s test and where sphericity could not be assumed epsilon corrections were utilised.

It was apparent that some participants could float with little or no movement whereas others required activity to maintain their airway clear of the water. Therefore, participants in Study 1 were classified as either “active” or “passive” floaters based on the arm and/or leg action during the last 30 s of their third float. “Active” floaters required arm or leg action to maintain their airway clear whereas “passive” floaters used limb movement only for stabilisation. The responses of these two groups were analysed separately to determine whether different instructions were required to improve their floating competency and the results are given in the supplementary material.

Relationships between anthropometric variables and floating competency (Float 1 – naïve and Float 3 – after float coaching) in still fresh water and still sea water (standard float after float coaching) were investigated using Spearman’s rank correlations.

Statistical analysis was performed using SPSS version 27 (IBM Corp, Armonk, NY, USA) and figures were produced using GraphPad Prism version 10 (GraphPad Software, Boston, MA, USA). Values are presented as mean (standard deviation) and statistical significance was taken as *P* ≤ 0.05.

## Results

### Participants

A total of twenty-five participants (11 women and 14 men) volunteered for the floats in different water conditions in Study 1, and 23 participants (10 women and 13 men) volunteered for the floats in the swimming flume in Study 2 (Table 1). The activities the participants undertook at the time of testing in and around water are shown in Table 2. Participants reported being either confident (*n* = 10) or very confident (*n* = 12) in water up to their chest (one of the inclusion criteria) and out of their depth their confidence averaged 4.0 (0.8) where 0 = very unconfident and 5 = very confident. All participants reported being capable of swimming 25 m in a heated swimming pool without assistance; reported swim ability was 6.2 (1.6) for participants in Study 1, 6.8 (1.1) for PRAC and 6.5 (1.1) for COACH (1 = “I cannot swim at all”; 10 = “I’m a really good swimmer”) and none of the participants were or had been competitive swimmers.

**Table 1:** Mean (standard deviation) participant characteristics and environmental conditions during the floats for Study 1 and 2. (W = women)

		**Study 1 (still open water)**		**Study 2 (indoor flume)**	
		Fresh water	Sea water	COACH	PRAC
Participant characteristics	N	22 (10 W)	13 (7 W)	11 (4 W)	12 (6 W)
	Age (y)	34.3 (10.1)	29.5 (10.3)	19.8 (1.1)	20.2 (1.2)
	Height (cm)	175.4 (8.7)	174.6 (9.0)	173.8 (8.8)	173.8 (7.9)
	Mass (kg)	80.8 (15.2)	81.6 (14.3)	67.7 (13.2)	70.7 (8.5)
	BMI (kg.m^−2^)	26.2 (4.4)	26.7 (3.9)	22.2 (2.6)	23.6 (3.7)
	Σ skinfolds (mm)	62.8 (22.9)	63.1 (16.7)	38.9 (14.0)	49.5 (21.6)
	Body fat (%)	26.8 (7.3)	27.2 (6.1)	18.0 (6.6)	21.6 (8.7)
	Waist:hip ratio	0.89 (0.05)	0.89 (0.04)	0.79 (0.06)	0.80 (0.06)
Environmental conditions	Air temperature (°C)	26.2 (3.7)	24.6 (3.7)	24.4 (1.6)	24.2 (1.3)
	WBGT (°C)	22.2 (2.4)	21.5 (2.4)	22.3 (1.7)	21.7 (2.0)
	Average wind speed (knots)	na	9.5 (5.4)		
	Gust speed (knots)	na	12.8 (4.9)		
	T_water_ 5 cm (°C)	22.9 (2.1)	20.9 (0.7)	31.1 (0.7)	31.6 (0.8)
	T_water_ 50 cm (°C)	22.5 (2.0)	20.7 (0.6)		
	Water flow (m.s^−1^)	0.0	0.0 (0.04)		
	Average wave height (cm)	na	6.9 (2.1)		
	Maximum wave height (cm)	na	12.2 (3.8)		
	Water specific gravity	1.000	1.027 (0.001)

**Table 2:** Water and swimming experience of participants. Note that the percentages do not add up to 100% as some participants reported undertaking water-based activities and swimming

	**Number and percentage engagement**		
	**Study 1** **(*n* = 25)**	**Study 2**	
**Activity/experience**		**PRAC** **(*n* = 11)**	**COACH** **(*n* = 12)**
No water-based activity	14 (56%)	2 (18%)	5 (42%)
Activities around (but not in or on) water	8 (32%)	8 (73%)	5 (42%)
Water-based activities	5 (20%)	1 (9%)	2 (17%)
Infrequently swim for recreation	4 (16%)	3 (27%)	0
Swim once a week for recreation	3 (12%)	0	1 (8%)
Swim regularly for recreation (2–3 times per week)	1 (4%)	0	0
Swim for fitness	1 (4%)	2 (18%)	0

Prior to any floats, participants were asked to describe the actions they would undertake to float and their responses are summarised in Table 3. The most common response from participants in Study 1 was to lie back but only 28% of these specified head back, the next most common response was to relax or stay calm. Eight participants reported arm/leg movement, three of which described sculling, four were vague and one suggested pushing down on water. Many thought the legs should be up on the surface and the core “engaged”, only five identified having arms/legs out as being necessary. Two participants stated breathing should be controlled and two said the lungs should be inflated.

**Table 3:** Initial perceived actions required to undertake a float prior to any floats or instruction. “Accurate” indicates correct action described (e.g. movement: sculling described), “Vague” indicates a response which may or may not be accurate (e.g. breathing: full lungs will increase buoyancy but is not sustainable) and “Hinder” indicates actions that are likely to hinder floating (e.g. pushing down on the water describes the incorrect action for sculling; engaging the core and keeping legs up will cause unnecessary effort and prevent relaxation). Number (percentage) of responses for each group are given (percentages do not add up to 100 %)

**n (%)**	**Study 1 (***n*** = 25)**			**Study 2: PRAC (***n*** = 12)**			**Study 2: COACH (***n*** = 11)**		
	Accurate	Vague	Hinder	Accurate	Vague	Hinder	Accurate	Vague	Hinder
**Head back**	5 (20 %)	13 (52 %)		2 (17 %)	4 (33 %)	2 (17 %)	1 (9 %)	5 (45 %)	4 (36 %)
**Relax**	9 (36 %)	3 (12 %)							
**Arms/legs spread**	7 (28 %)			6 (50 %)			4 (36 %)		1 (9 %)
**Movement**	3 (12 %)	4 (16 %)	1 (4 %)		6 (50 %)		1 (9 %)	5 (45 %)	
**Breathing**	4 (16 %)	2 (8 %)		1 (8 %)			1 (9 %)	1 (9 %)	
**Engage core**			5 (20 %)			3 (25 %)			4 (36 %)
**Legs up**			7 (28 %)			1 (8 %)			

In Study 2, all participants reported attempting to float previously and this was, on average, 1.7 (3.0) years ago (range 1 month to 10 years) for the PRAC group and 3.2 (5.2) years ago (range 1 week to 13 years) for the COACH group. In both groups, approximately half of the participants stated you should lie back to float, only 3 (2 in PRAC, 1 in COACH) specified head back and 6 (2 in PRAC, 4 in COACH) said head up or out of water (Table 3). Approximately half of the participants reported arm/leg movement, although these were generally vague with only 1 participant describing sculling. Six participants in PRAC and four in COACH reported a starfish shape, although one participant in COACH stated the arms should be at the side. Several participants (3 in PRAC, 4 in COACH) reported the core should be engaged, in PRAC one participant thought the legs should be kept up whilst in COACH one participant thought the legs should be down. None of the participants in Study 2 reported relaxing as being necessary for floating.

### Study 1: Still fresh water floats

**Fig. 2:**
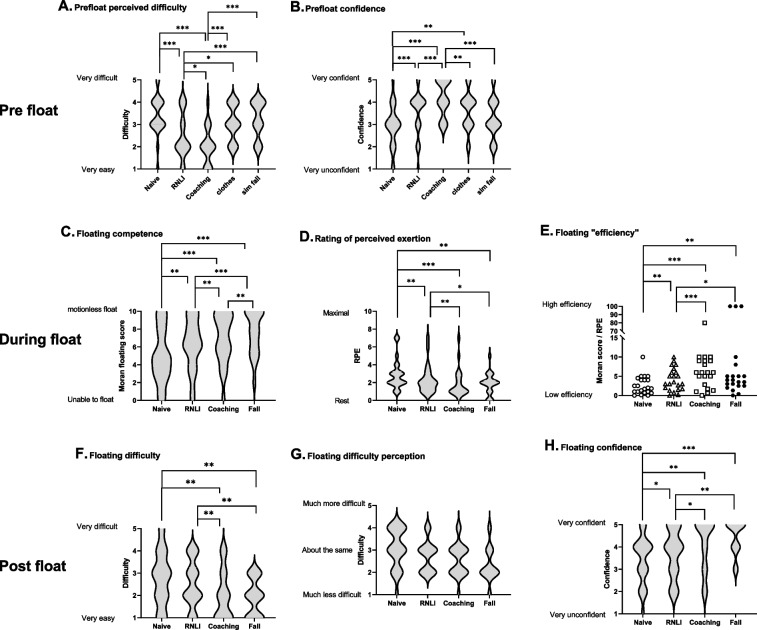
Responses before (Pre float), during and after (Post float) floats in fresh water (*n* = 22). The width of the violin plot indicates the frequency distribution. Bars indicate significant differences between floats * *P* < 0.05, ** *P* < 0.01 and *** *P* < 0.001

Participant characteristics and environmental conditions are shown in Table 1. Perceived floating difficulty prior to the float decreased over the first three floats (Float 1: 3.4 [0.9], Float 2: 2.5 [1.0], Float 3: 2.0 [0.8]; *P* < 0.05, Fig. 2A). Float 4 was expected to be more difficult than either Float 2 (*P* < 0.05) or Float 3 (*P* = 0.001) with the perceived difficulty expected with clothing and the simulated fall being similar (3.1 [0.8] vs 3.2 [0.8], *P* = 0.417).

Confidence prior to the floats increased over the first three floats (Float 1: 2.9 [1.0], Float 2: 3.5 [1.0], Float 3: 4.2 [0.8]; *P* < 0.01, Fig. 2B). Participants felt less confident prior to Float 4 compared to Float 3 (*P* < 0.01) with the impact of clothing and the simulated fall being the same (3.6 [0.8] and 3.3 [0.9] respectively). Confidence in floating with clothes during Float 4 was greater than prior to Float 1 (*P* = 0.004).

Floating competence, as assessed using the Moran scale, improved with each float (Float 1: 5.0 [3.1], Float 2: 6.3 [2.9], Float 3: 6.9 [2.8], Float 4: 7.6 [2.4]; *P* < 0.01, Fig. 2C), with less movement required to keep the airway clear with each successive float. Although most participants with a Moran score of 8 or above floated horizontally on the surface, there were some participants who could float with minimal movement in a more vertical position. This was associated with a reduction in perceived exertion (RPE) with successive floats (Float 1: 2.9 [1.7], Float 2: 2.3 [1.6], Float 3: 1.9 [1.6], Float 4: 1.8 [1.2]; *P* < 0.05, Fig. 2D). However, although RPE during Float 4 was lower than Float 1 and 2, it was not different from Float 3. Combining the Moran scores and RPE scores to get an index of floating efficiency showed that participants became more efficient with successive floats (Float 1: 2.6 [2.4], Float 2: 4.2 [2.9], Float 3: 9.1 [16.6], Float 4: 17.5 [34.6]; *P* < 0.05, Fig. 2E), though no further improvement was seen between Floats 3 and 4.

Compared to Float 1 and 2 (2.8 [1.2] and 2.5 [1.1] respectively), participants reported floating was easier during Float 3 and 4 (2.0 [1.1] and 1.9 [0.8] respectively; *P* < 0.01), however there was no difference in perceived difficulty between Floats 1 and 2 or Floats 3 and 4 (Fig. 2F). For all floats, participants reported floating being between “about the same” and “less difficult” than they thought (Fig. 2G). Confidence in floating ability increased with successive floats (Float 1: 3.4 [1.1], Float 2: 3.7 [1.8], Float 3: 4.1 [1.2], Float 4: 4.5 [0.7]; *P* < 0.05, Fig. 2H), though confidence did not significantly increase between Floats 3 and 4.

The number and percentage of participants that reported their breathing on immersion interfered with their ability to float is shown in Table 4. Interference from the cold shock decreased over the first three immersions (*P* < 0.05), but increased during Float 4 compared to Float 3 (*P* = 0.038) due to head immersion during the simulated fall. Less than 25% of participants reported any interference from shivering and no significant difference was observed between floats (Table 4).

**Table 4:** Number (percentage) of participants reporting breathing and shivering affecting their ability to float at the start of their immersion in water at 23 °C

		**Float 1**	**Float 2**	**Float 3**	**Float 4**
		**Naive**	**RNLI**	**Coaching**	**Fall**
Breathing	Not at all	7 (32%)	13 (59%)	14 (67%)	11 (50%)
	A little bit	9 (41%)	4 (18%)	7 (33%)	7 (32%)
	Quite a lot	3 (14%)	4 (18%)	0	3 (14%)
	Greatly affected	2 (9%)	1 (5%)	0	1 (5%)
	Breathing stopped me floating	1 (5%)	0	0	0
Shivering	Not at all	17 (77%)	17 (77%)	18 (86%)	17 (77%)
	A little bit	3 (14%)	1 (5%)	3 (14%)	3 (14%)
	Quite a lot	2 (9%)	4 (18%)	0	2 (9%)

The majority of participants (64%) found the RNLI messaging helpful. After watching the RNLI “Float to Live” video and looking at the associated poster, 7/22 participants reported the instruction to “put your head back” helped them to float (Table 5). 4/22 participants found putting their arms or legs out helped. 3/22 of participants reported the instructions to relax, control breathing or move their limbs helped. Two thought that practice had helped them. On the other hand, two reported that keeping their legs up was useful, however for these two individuals this probably increased the effort required as they naturally floated in a vertical position. Four participants did not find the instructions helped their float and four reported they were confusing or unhelpful (“*video didn't help, concentrated too much and didn't relax”; “image—bent knees & let bum sink”; “Move arms and legs—vague & not helpful”; “Use arms and legs—didn't help how should they be used? Swim and shout for help counter to previous floating advice”*).

**Table 5:** Instructions from the RNLI video and poster, following float coaching and following the last float (Sim fall), that the participants reported helping their floating. Number (percentage) of responses are given for all participants and those considered active or passive floaters

	**Still fresh water**									**Sea water**			
	All (*n* = 22)			Active floaters (*n* = 9)			Passive floaters (*n* = 13)			Still (*n* = 13)		Moving	
	RNLI video	Float coaching	Sim fall	RNLI video	Float coaching	Sim fall	RNLI video	Float coaching	Sim fall	Float coaching	Sim fall	Float 1 (*n* = 6)	Sim Fall (*n* = 4)
**Head back**	7 (32%)	10 (45%)	10 (45%)	4 (44%)	3 (33%)	5 (56%)	3 (23%)	7 (54%)	5 (38%)	7 (54%)	7 (54%)	5 (83%)	3 (75%)
**Arms/legs out**	4 (18%)	4 (18%)	3 (14%)	1 (11%)	1 (11%)		3 (23%)	3 (23%)	3 (23%)	4 (31%)	2 (15%)	1 (17%)	1 (25%)
**Relax**	3 (14%)	10 (45%)	12 (55%)		4 (44%)	4 (44%)	3 (23%)	6 (46%)	8 (62%)	5 (38%)	7 (54%)	3 (50%)	4 (100%)
**Breathing**	3 (14%)	9 (41%)	7 (32%)		2 (22%)	2 (22%)	3 (23%)	7 (54%)	5 (38%)	4 (31%)	5 (38%)	2 (33%)	2 (50%)
**Limb movement**	3 (14%)	10 (45%)	6 (27%)	2 (22%)	7 (78%)	4 (44%)	1 (8%)	3 (23%)	2 (15%)	1 (8%)	1 (8%)	1 (17%)	
**Practice**	2 (9%)		1 (5%)				2 (15%)		1 (8%)			1 (17%)	1 (25%)
**Leg position**	2 (9%)	6 (27%)	6 (27%)	2 (22%)	3 (33%)	3 (33%)		2 (15%)	3 (23%)	4 (31%)	1 (8%)	3 (50%)	3 (75%)
**None**	4 (18%)	1 (5%)	1 (5%)	2 (22%)			2 (15%)	1 (8%)	1 (8%)				
**Unhelpful**	4 (18%)						3 (23%)						

The most helpful instructions provided by the swim instructors were to put their head back further (so the ears were submerged), relax and not to hold their breath but to keep breathing (Table 5). Instructions on limb action were also helpful and these included: “*stroking the dog, kicking a little”; “hands in sideways motion rather than up and down”; “sculling deeper, kicking legs”*. Being told it didn’t matter if their legs sank helped four participants and not having to be in a starfish position also helped another participant.

On the final float, the most frequent helpful instructions reported by the participants were to relax and put their head back (Table 5). Other comments included: “*not worrying if legs sink”; “stroke cat/dog lower in water”; “calm breathing as quickly as possible”.*

### Study 1: Still sea water floats

**Fig. 3:**
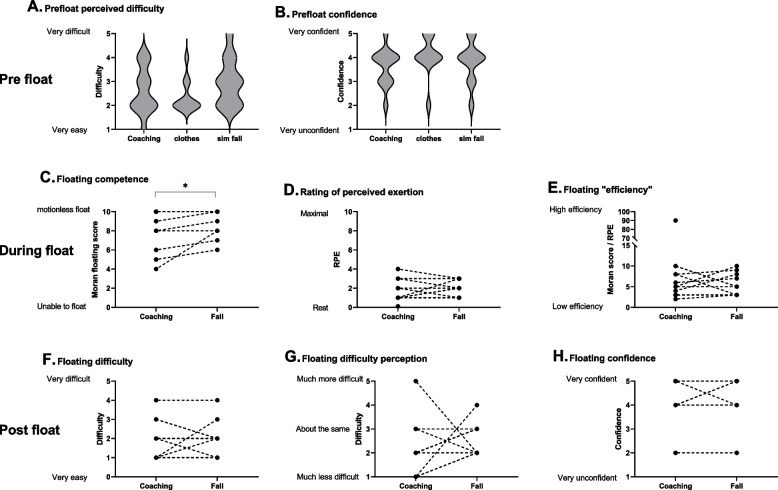
Individual responses of the participants who undertook floats in still sea water. 10 participants had coaching and previous floating practice (4 floats) in still fresh water and 3 participants had coaching and previous floating practice (2 floats) in still sea water. The width of the violin plot indicates the frequency distribution (the wider the violin, the more participants reported this score and vice versa), * *P* < 0.05

Participant characteristics and environmental conditions are shown in Table 1. Three participants undertook all four floats (1. naïve, 2. following RNLI instruction, 3. following float coaching from a swim instructor and 4. a simulated fall) in still sea water; their responses are given in the supplementary material. The responses to floats following coaching either in fresh water or sea water and following a simulated fall are shown in Fig. 3. Floating competence improved as less movement was required in the final float (8.1 [2.1] vs 8.9 [1.4]; *P* = 0.034, Fig. 3C simulated fall), however this was not accompanied by any change in perception of the difficulty or confidence in floating either prior to (Fig. 3A and B) or after the float (Fig. 3F to H).

Instructions that helped the participants to float are shown in Table 5. Those most frequently reported were: head back (one participant stated *string on back of head instruction*), relax or stay calm and control breathing. Having the arms or legs wide and adjusting them to the wave motion was also reported as helpful. Eight participants indicated that they would have benefitted from knowing how to deal with waves e.g. “*how to relax/breathe with wave splash over face*”. One participant reported that it would be helpful if information on “*the difference between floating in sea and fresh water*” was given as they did not know they were more buoyant in sea water, this was probably the case with other participants but it was not reported.

#### Fresh vs sea water floats

Ten participants (6 women, 4 men; height: 176.3 [8.8] cm; mass: 84.5 [15.8] kg; BMI: 27.0 [4.3] kg.m^−2^; sum of skinfolds: 62.0 [15.4] mm; body fat: 28.2 [5.2]; waist:hip ratio: 0.90 [0.04]) undertook floats in still fresh water and sea water.

**Fig. 4:**
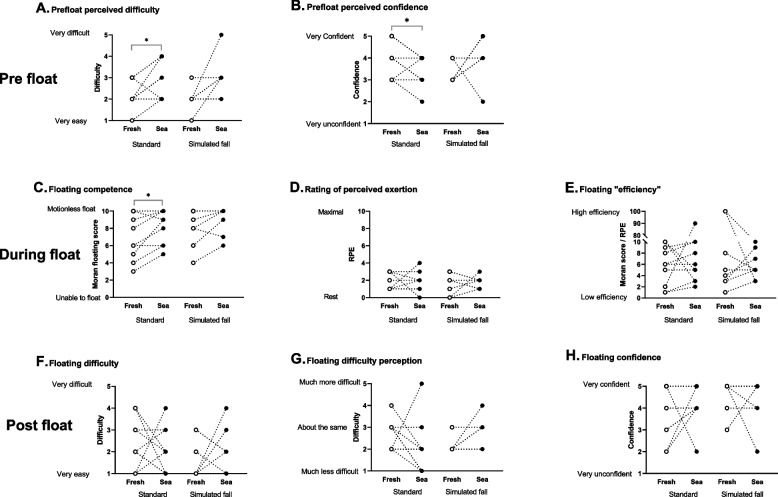
Comparison of floats in still fresh water and sea water. Individual responses are shown for the standard floats (wearing swim wear—Float 3 in fresh water vs float 1 in sea water; *n* = 10) and floats following a simulated fall (wearing shorts and T-shirt—Float 4 in fresh water vs float 2 in sea water *n* = 8), * *P* < 0.05

Prior to their still water floats, participants perceived floating in sea water would be more difficult than in fresh water (2.8 [0.8] vs 2.2 [0.6]; *P* = 0.035, Fig. 4A) and had less confidence (3.7 [0.9] vs 3.6 [0.7]; *P* = 0.034, Fig. 4B). Although their floating competence was better in sea water (8.6 [1.8]) compared to fresh water (7.3 [2.4]; *P* = 0.031, Fig. 4C) this was not reflected in their reported perception of effort (Fig. 4D), difficulty (Fig. 4F and G) or confidence (Fig. 4H) which did not differ between conditions. When asked which condition they thought was easiest to float in, most participants stated sea water was easier (8/10). Two participants (both with a Moran score of 10 in fresh water) found fresh water floats easier due to wave action in the sea. No differences were observed between the floats in fresh water or sea water following a simulated fall (Fig. 4).

#### Anthropometric factors influencing floating competence

**Fig. 5:**
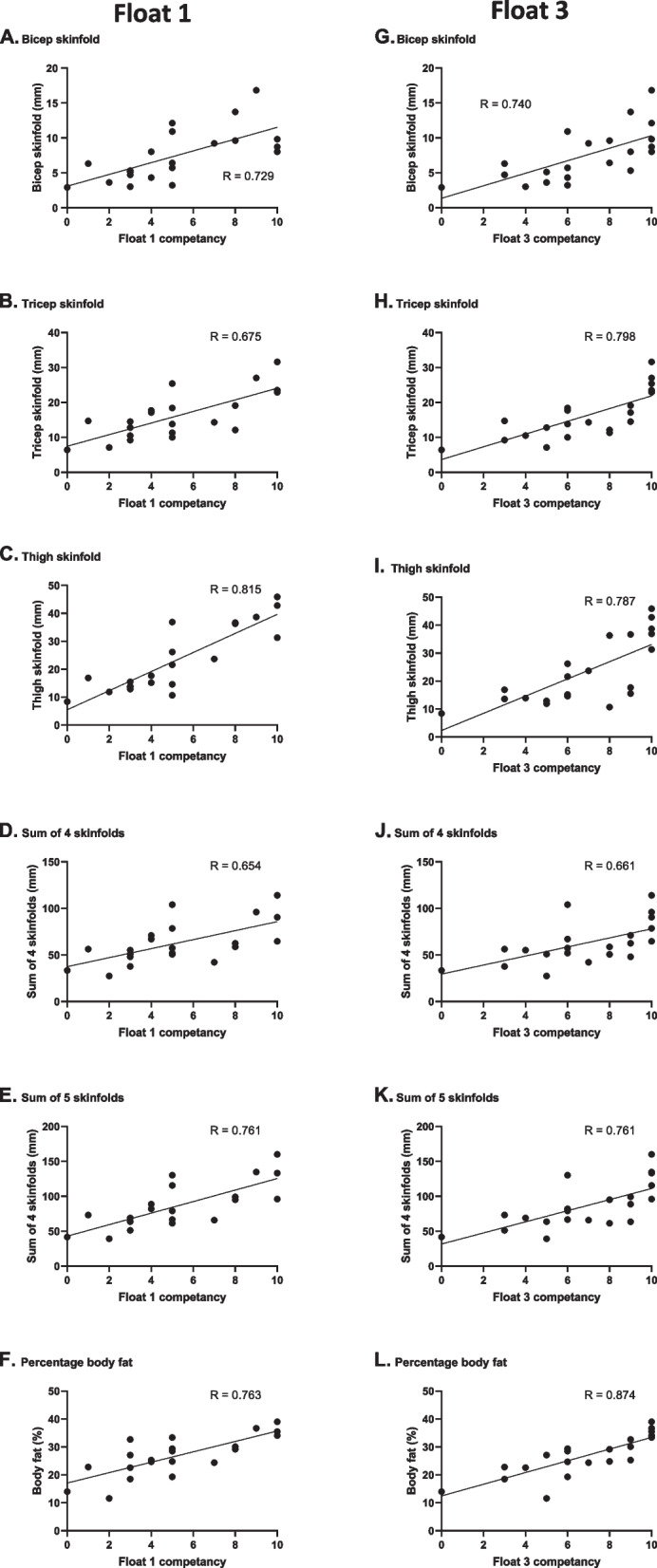
Correlations between anthropometric measures and floating competence in still fresh water in Float 1 (naïve, **A** to **F**) and Float 3 after instruction (**G** to **L**). Individual data points, regression line and R value are shown (*n* = 21)

Correlations were conducted between floating competence as assessed using the Moran scale and participants’ anthropometric measurements. In still fresh water, floating competence during the first float was positively correlated with skinfold thickness at the biceps (*P* < 0.001; Fig. 5A), triceps (*P* = 0.003; Fig. 5B) and thigh (*P* < 0.001; Fig. 5C) as well as the sum of 4 skinfolds (biceps, triceps, subscapular and supra iliac; *P* = 0.001; Fig. 5D), sum of 5 skinfolds (biceps, triceps, subscapular, supra iliac and thigh; *P* < 0.001; Fig. 5E) and percentage body fat (*P* < 0.001; Fig. 5F).

Floating competence during Float 3 (after instruction) in still fresh water was correlated with the same variables as seen in Float 1: biceps skinfold (*P* < 0.001; Fig. 5G); triceps skinfold (*P* < 0.001; Fig. 5H); thigh skinfold (*P* < 0.001; Fig. 5I); sum of 4 skinfolds (*P* = 0.001; Fig. 5J), sum of 5 skinfolds (*P* < 0.001; Fig. 5K) and percentage body fat (*P* < 0.001; Fig. 5L). No significant correlations were observed with mass, BMI, waist:hip ratio, subscapular skinfold or supra iliac skinfold and floating competency for either Float 1 or Float 3.

**Fig. 6:**
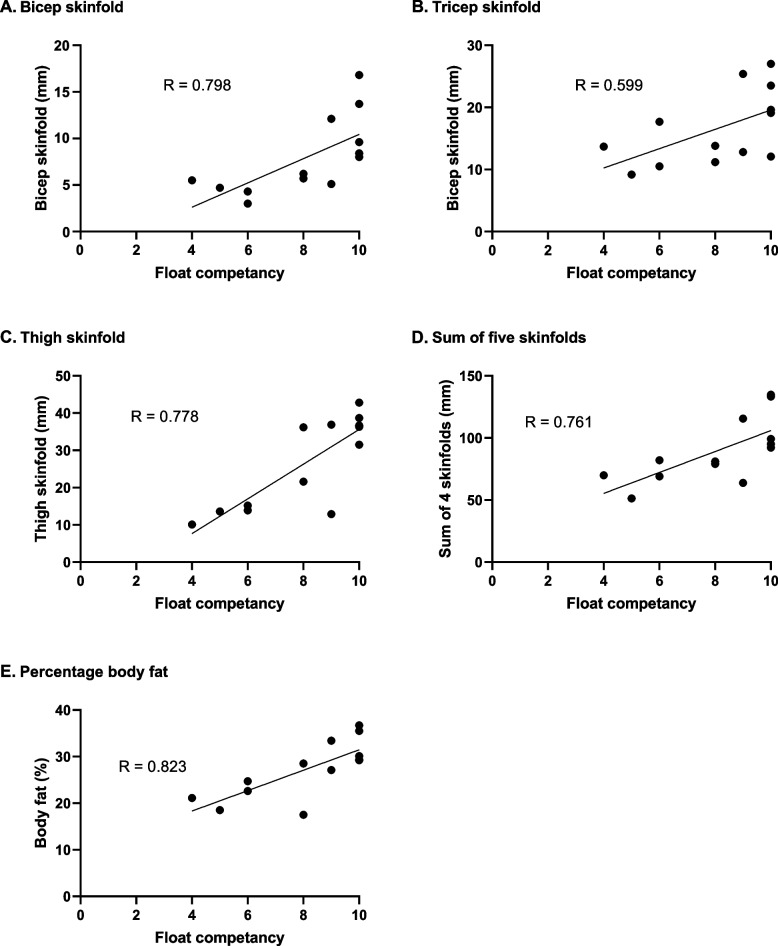
Correlations between anthropometric measures and floating competence in still sea water after instruction in either fresh or sea water. Individual data points, regression line and R value are shown (*n* = 13)

Fewer relationships were observed between floating competency in still sea water (following instruction in either fresh water or sea water) and anthropometric measures. As with the floats in still fresh water, correlations were observed between biceps skinfold (*P* = 0.003; Fig. 6A), triceps skinfold (*P* = 0.031; Fig. 6B), thigh skinfold (*P* = 0.005; Fig. 6C), sum of 5 skinfolds (*P* = 0.007; Fig. 6D) and percentage body fat (*P* = 0.002; Fig. 6E). No significant correlations were observed with any of the other measures.

### Study 2: Coaching versus practice

**Fig. 7:**
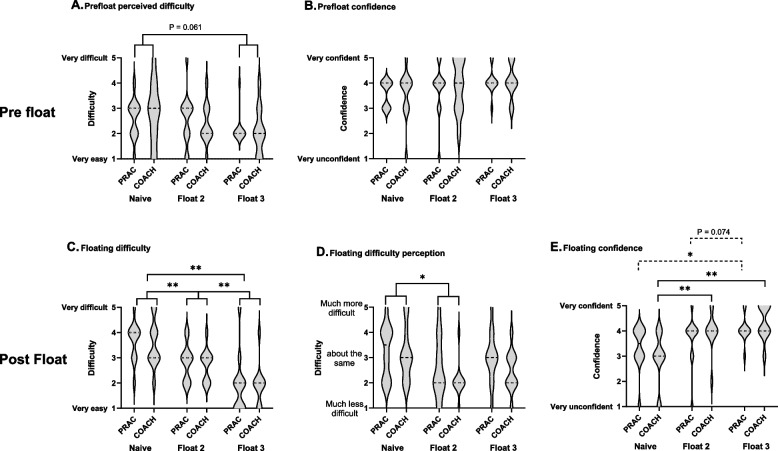
Pre and post float perceived floating difficulty and confidence for PRAC (*n* = 11) and COACH (*n* = 12) groups floating in a flume in Study 2. The width of the violin plot indicates the frequency distribution and the dashed line the median. Bars indicate significant differences between floats * *P* < 0.05, ** *P* < 0.01 (Panel E: solid bars show differences between floats for COACH and dashed bars show differences between floats for PRAC)

Participant characteristics and environmental conditions are shown in Table 1. Prior to floating, there was no difference in perceived float difficulty or confidence between PRAC and COACH (Fig. 7A and B). There was a trend for a main effect of float with perceived difficulty decreasing between Float 1 (naïve) and Float 3 (2.7 [1.0] vs 2.1 [0.8], *P* = 0.061).

**Fig. 8:**
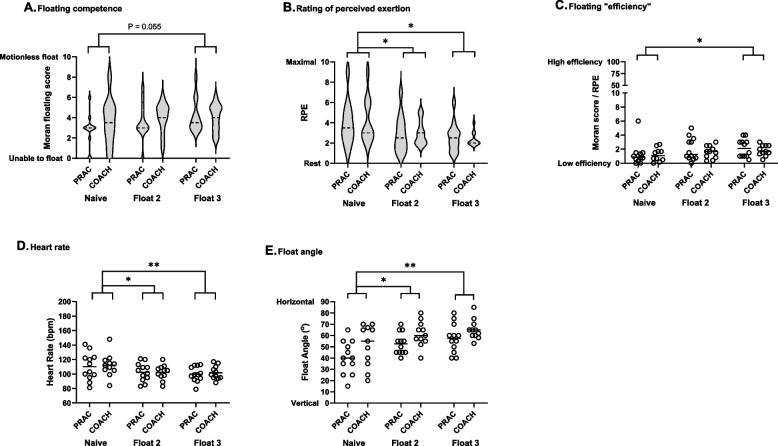
Responses to floats in the flume for PRAC (*n* = 11) and COACH (*n* = 12) groups in Study 2. The width of the violin plot indicates the frequency distribution and the dashed line the median. Bars indicate significant differences between floats * *P* < 0.05 and ** *P* < 0.01

No differences were observed between PRAC and COACH for floating competency (Fig. 8A), RPE (Fig. 8B), efficiency (Fig. 8C), heart rate (Fig. 8D) and angle (Fig. 8E). However, there was a main effect of float, with float efficiency improving between Float 1 and 3 (1.2 [1.3] vs 1.9 [1.0]; *P* = 0.047; Fig. 8C); float angle becoming more horizontal between Float 1 and 2 (45 [17] ° vs 57 [11] °; *P* = 0.005) and Float 1 and 3 (45 [17] ° vs 61 [12] °; *P* = 0.006; Fig. 8E); heart rate decreasing between Float 1 and 2 (111 [17] bpm vs 102 [11] bpm; *P* = 0.020) and Float 1 and 3 (111 [17] bpm vs 101 [10] bpm; *P* = 0.006; Fig. 8D); and RPE decreasing between Float 1 and 2 (4.0 [2.1] vs 3.0 [1.5]; *P* = 0.014) and Float 1 and 3 (4.0 [2.1] vs 2.5 [1.1]; *P* = 0.001; Fig. 8A). There was also a trend for float competency to improve between Float 1 and 3 (3.1 [[2.0] vs 4.0 [1.4]; *P* = 0.055; Fig. 8A).

Post float difficulty was not different between groups and reduced with successive floats (Float 1 = 3.6 [0.8], Float 2 = 2.8 [0.7] and Float 3 = 1.9 [1.0]; *P* = 0.001; Fig. 7C). Participants also reported finding the float less difficult than they thought in Float 1 compared to Float 2 (3.2 [1.0] vs 2.4 [1.0]; *P* < 0.001; Fig. 7D). Post float confidence was increased with successive floats (Float 1 = 3.1 [1.0], Float 2 = 3.8 [0.9] and Float 3 = 4.2 [0.6]; Float 1 vs 2 and 3, *P* < 0.001; Float 2 vs 3, *P* = 0.015; Fig. 7E). There was an interaction effect between float and group with COACH increasing their confidence after their 2nd float (Float 1 = 2.9 [1.0], Float 2 = 4.0 [0.8] and Float 3 = 4.4 [0.7]; Float 1 vs 2 and 3, *P* < 0.001; Float 2 vs 3, *P* = 0.168) and PRAC increasing their confidence after their 3rd float (Float 1 = 3.3 [0.9], Float 2 = 3.7 [1.0] and Float 3 = 4.1 [0.5]; Float 1 vs 2, ns; Float 1 vs 3, *P* = 0.026; Float 2 vs 3, *P* = 0.074). However, there was no between group difference.

## Discussion

This is the first study to evaluate the effectiveness of a public safety campaign on floating competence of inexperienced water users in realistic open water conditions. The RNLI messaging did improve float competence and confidence whilst reducing perceived difficulty. However, further improvements were made following individualised float coaching indicating the messaging could be refined further especially for active floaters. Importantly these instructions were applicable in a range of realistic open water scenarios (open fresh and sea water, slow moving water, with clothing and following a simulated fall) and therefore we can accept our hypothesis.

The participants who volunteered for the current studies were not experienced water users and none were trained or competitive swimmers and only 32% were confident in their ability to float prior to taking part. Participants in Study 1 were men and women from a wide age range (20 to 60 years old) with a similar BMI to the adult UK population (27.6 kg.m^−2^;) [13] and therefore representative of the general adult population regarding their physical characteristics.

Floating competence in still fresh water was improved with the combination of instruction and practice. This was associated with an improved float efficiency and decreased RPE. As a consequence, perceived floating difficulty decreased and confidence increased. The RNLI messaging helped most of the participants (64%) to float, with the instruction to “put your head back” being reported the most helpful (Table 5). The swim teachers were able to emphasise this body position by giving more specific instructions (put head back so the ears are submerged) which was helpful to additional participants (Table 5). This corresponds to backstroke swimming where inexperienced observers did not fully appreciate the importance of the correct head position compared to experienced swim teachers [18]. From the RNLI messaging, many of the participants interpreted that a horizontal float position should be adopted and as a result some expended considerable energy keeping their legs up. Being told by the swim teachers that it is OK if their legs dropped was found to be helpful, particularly for the participants who naturally floated in a more vertical position. One of the most frequent instructions given by the swim teachers was to relax, interestingly participants seemed to report this as more helpful the more floating practice they had, with it helping 45% on their 3rd float, 55% on their 4th float (simulated fall with clothing), with all of the participants finding it helpful following a simulated fall into moving sea water (after 6 to 8 floats; Table 5).

The RNLI messaging to “use your arms and legs to keep you afloat” was not found helpful and if done incorrectly would actually impede floating. The participants who were “active” floaters benefitted from the additional instructions given by the swim teachers describing sculling. For some participants, the analogy of “stroking a dog” to describe the sculling action was particularly helpful. The use of analogies has been found to facilitate implicit learning [10, 23] and enhance motor skill acquisition in swimming whilst still enabling learners to develop individual movement patterns [8]. Therefore, combining implicit (sculling analogy) and explicit (head position) instructions is important as the required floating technique is individual and dependent on anthropometric characteristics as well as environmental conditions. Furthermore, a motor skill developed through implicit learning is less affected by stress [9, 10] and retained for longer [1], both factors being important in the event of an accidental immersion. In the current study, floating competence was not impaired (and actually improved) following a simulated fall despite participants feeling less confident prior to the float. This could reflect the robustness of the prior implicit learning which was unaffected by the stress associated with head submersion.

Whilst we did not measure skill retention specifically, 10 participants undertook floats in sea water 25 days (range 2 to 54 days) after being coached in fresh water and no difference in float competence was observed indicating floating skills had been retained. The inclusion of analogies to promote implicit learning may be beneficial in public messaging as it is less affected by intelligence than explicit learning [16] and will therefore be useful to a wider audience.

The skills learnt in still water enabled successful floats in moving water (see supplementary material) indicating that the floating skills learnt in a pool will be transferrable to a wide range of open water environments. Where water is turbulent, aerated or obstacles are present, defensive floating would be more appropriate. However even in the moving fresh water representative of a Grade 1 river, participants were able to float successfully. Whilst these results are encouraging, the number of participants undertaking floats in the moving water was small (*n* = 5 for fresh water and *n* = 6 for sea water) thus limiting the conclusions that can be drawn.

Participants perceived that wearing clothing would make floating more difficult. However, despite the worst-case scenario of a vertical drop into the water (which removes most of the inherent buoyancy provided by clothing) floating competency was found to further improve. This is similar to previous research [21], where despite clothing increasing buoyancy it was perceived to increase the difficulty of floating by “dragging people down”. Another misconception was that it would be harder to float in the sea, with some participants not knowing that they would be more buoyant in salt water. Whilst less activity was required to maintain a float in sea water compared to fresh water for active floaters (Fig. 3), participants who were motionless in the still lake needed to undertake some activity in the sea to stabilise themselves in the slight chop.

The second study was conducted to examine the relative role of practice and instruction on floating skill acquisition. Unlike the study in open water, no statistically significant improvements in floating competence (using the Moran score) were observed after practice, instruction or a combination practice and instruction (Fig. 8). However, heart rate was reduced and this was accompanied by a reduction in perceived effort and difficulty and increased confidence (Figs. 7 and 8). The lack of effect of either training or practice on floating competence in Study 2 is probably due to combination of the situation and the poor sensitivity of Moran score to measure floating competence. The restricted size of the flume meant that participants who floated vertically had to actively raise their legs and the actions of some participants set up a standing wave which increased the difficulty of the float. Although the results did not support our hypothesis, that coaching would improve floating competence more than practice, 86% participants stated a preference for coaching followed by practice as this increased their confidence level early and provided an opportunity to practice later.

Correlations between body composition and floating competency (Figs. 5 and 6) indicate that higher levels of subcutaneous body fat were advantageous to floating as they reduced the activity required to maintain a float. This finding is unsurprising given the buoyancy afforded by body fat. Previous studies have demonstrated that airway freeboard during floating is correlated with both sum of skinfold thickness and percentage body fat [2]. Neither BMI nor waist to hip ratio acted as surrogates for body fat measurement as no correlations were found between them and floating competency. This is not surprising since BMI does not distinguish between muscle and fat mass whereas fat, but not muscle, increases buoyancy. Therefore, simple measures (such as BMI or girth measurements) that the general population could do on themselves would not be reliable indicators of their buoyancy and, therefore, whether they are likely to be “active” or “passive” floaters. Further research is required to develop a comprehensive floating topography from recording the range of actions and positions which enable successful floats in individuals encompassing a wide range of body compositions and anthropometry.

Despite the temperature of the open water averaging 23 °C, 68% of participants found that breathing affected their ability to float to lesser or greater extent. It is a commonly held misconception that the cold shock response is only seen in water temperatures of 15 °C or below [25], in fact the maximum response is evoked between 10 °C and 15 °C [20]. Therefore, any messaging should make the public aware that even in open water usually considered “warm”, individuals may experience the cold shock and that this will impact on their ability to breath hold and float. In colder water, both the magnitude and duration of the cold shock response would be increased [20] and this may impair the ability to float. As expected interference from the cold shock decreased over the first three immersions (*P* < 0.05), but increased during Float 4 compared to Float 3 (*P* = 0.038) due to head immersion during the simulated fall. This reduction in response is due to habituation of the cold shock response with repeated immersions [6] and can occur even when immersions occur on the same day [5].

Less than 25% of participants reported any interference from shivering during their floats (Table 4) and this was due to the relatively warm water and the short duration of immersion. In colder water, when there is greater skin cooling and during prolonged immersions (where core cooling will also occur) the shivering response will be much greater [22]. The combination of the behavioural response to curl up (to reduce the surface area for heat loss) and intensive shivering is likely to reduce the ability to float.

## Conclusions

This study has demonstrated that a public health campaign “Float to Live” can improve the floating competence and confidence of inexperienced water users in realistic open water scenarios. Further improvements were observed following additional instructions provided by swim teachers. As a result, “tilt your head back with ears submerged”, “relax and move your hands to help you stay afloat” and an image of an individual floating in a more vertical position have now been incorporated into the new RNLI “Float to Live” messaging. Importantly, the skills acquired were applicable in a range of realistic open water scenarios (open fresh and sea water, slow moving water, with clothing and following a simulated fall) and were retained. Given there were no differences in floating competence between the COACH and PRAC groups, individuals should practice floating as the skills learnt are transferable to a range of open water conditions.

### Supplementary Information

Supplementary Material 1.

Supplementary Material 2.

Supplementary Material 3.

## Data Availability

The anonymised data collected are available as open data via the University of Portsmouth online data repository.

## Abbreviations

ANOVA: Analysis of variance
CIWWC: Cardiff International White Water Centre
COACH: Group undertaking floats in following order: 1) naïve, 2) after coaching, 3) after practise
CSR: Cold shock response
PRAC: Group undertaking floats in following order: 1) naïve, 2) after practise, 3) after coaching
RNLI: Royal National Lifeboat Institute
RPE: Rating of perceived exertion
SLS-GB: Surf Life Saving GB
SUP: Stand up paddleboard
