# Supplementary material for: Inexperienced water users can “Float to Live” in realistic open water conditions
Source: BMC Public Health. 2024 Jul 29;24:2030. doi: 10.1186/s12889-024-19409-6 (PMC11285178; doi:10.1186/s12889-024-19409-6)
Supplement: Supplementary file 1 — Supplementary Material 1. [file 12889_2024_19409_MOESM1_ESM.docx]

**Inexperienced water users can “Float to Live” in realistic open water conditions**

Clare Eglin, Heather Massey, Geoff Long, Adrian Mayhew and Michael Tipton

**Annex 1: Active vs Passive floating in still fresh water (Study 1)**

*“Active” Floaters in still fresh water*

Nine participants (all men; height: 181.4 [4.5] cm; mass: 87.0 [11.7] kg; BMI: 26.4 [3.1] kg.m^-2^; sum of skinfolds: 54.5 [22.6] mm; body fat: 21.3 [6.0]; waist:hip ratio: 0.90 [0.06]) were considered “active” floaters based on the arm and/or leg action required to maintain their airway clear.

All participants showed an improved floating competence with practice/instruction (P < 0.05, Figure A1.1A). One participant did not improve until Float 4 because they were not in deep enough water for their natural vertical position in the previous floats. RPE decreased and floating “efficiency” increased with successive floats (P < 0.05) except for between Float 3 and 4 where there was no difference (Figure A1.1B and C). Perceived floating difficulty decreased with successive floats (P < 0.05, Figure A1.1D) and confidence in ability to float increased (P < 0.05, Figure A1.1E) although there was no further improvement between Floats 3 and 4.

Following watching the RNLI “Float to Live” video and looking at associated poster, 4/9 participants reported the instruction to lie back helped them to float (Table A1.1). Two found moving their arms/legs helped and one having their arms out, though they reported “*this didn’t seem to help”*. Two reported that keeping their legs up was useful, however for these two individuals this probably increased the effort required as they naturally floated in a vertical position. Two participants did not find the instructions helped their float.

The instructions provided by the swim teachers and the corresponding instructions the “active” floaters found helpful are given in Table A1.1. The most helpful instructions were regarding limb movement and included: “*stroking the dog, kicking a little”; “hands in sideways motion rather than up and down”; “sculling deeper, kicking legs”; “stroke cat - not up and down, kick from hips & keep legs straight”; “arms side to side (felt effortless)”* (Table A1.1)*.* Other frequently reported helpful instructions were relax, head back and don’t worry about legs dropping. On the final float, the most frequent helpful instructions reported by the participants were to relax, put their head back and undertake the correct limb movement (Table A1.1). Not worrying if their legs sank and controlling their breathing also helped.

*“Passive” floaters in fresh water*

13 participants (3 men, 10 women: height: 173.5 [8.6] cm; mass: 75.8 [16.7] kg; BMI: 25.6 [5.2] kg.m^-2^; sum of skinfolds: 66.5 [21.2] mm; body fat: 30.5 [4.8]; waist:hip ratio: 0.89 [0.04]) were considered “passive” floaters based on the arm and or leg action they used was for stabilisation rather than to maintain their airway clear.

Floating competence was not significantly improved after watching the RNLI video (P = 0.057; Figure A1.2A), although one participant went from treading water (Moran score 0) to an almost motionless float (Moran score 9). Floating competence was significantly improved during Float 3 and 4 compared to Float 1 and 2 (Figure A1.2A). RPE decreased in the final 2 floats compared to the first float (Figure A1.2B) and floating “efficiency” increased from Floats 1 and 2 to Float 3 and 4 floats (P < 0.05; Figure A1.2C). Perceived floating difficulty did not change significantly across each of the floats (Figure A1.2D), confidence in ability to float did however increase and was greatest in Float 3 and 4 (Figure A1.2E).

Following watching the RNLI Float to Live video and looking at associated poster, 5/13 participants reported the instructions either did not help or were unhelpful (“*image - bent knees & let bum sink”; “Move arms and legs - vague & not helpful”; “Use arms and legs - didn't help how should they be used? Swim and shout for help counter to previous floating advice”*). Helpful instructions included to put your head back, have your arms/legs out, relax and control breathing but these were only reported by 3/13 participants (Table A1.2). Two reported practice was helpful.

The instructions provided by the swim teachers and the corresponding instructions “passive” floaters found helpful are given in Table A1.2. The most helpful instructions provided by the swim instructors were to put their head back, control breathing (“*breathing properly”; “keep breathing”; “control breathing”; “breathe more”; “focus on breathing especially when shivering”; “more relaxed breathing”; “not breath holding”; “regular breathing”*) and relax (Table A1.2). Helpful instructions regarding limb movement were more passive in nature and included: “*trying not to move limbs as much”; “slower hand movement”; “sideways arm movement”*. Helpful instructions for limb position included: “*don't try mimic position in video”; “don't worry about legs if they drop”; “keeping hips up and level”; “not worry about legs”*; *“OK to float in a different position from starfish, legs wider to give more stability”* (this participant floated with their hands behind their head).

On the final float, the most frequent helpful instructions reported by the participants were to relax, put their head back and controlling their breathing (Table A1.2). Not worrying if their legs sank and adjusting the position of their arms (“*palms down not as stable as relaxed position [thumbs up]”; “hands behind neck”; “palms down”*) also helped.

**Figure A1.1.** Individual responses of participants considered “active floaters” for each of their 4 floats in still fresh water (n = 9). Grey bars indicate significant differences between floats * P < 0.05 and ** P < 0.01.

**Figure A1.2.** Individual responses of participants considered “passive floaters” for each of their 4 floats in still fresh water (n = 12). Grey bars indicate significant differences between floats * P < 0.05 and ** P < 0.01

**Table A1.1.** Floating instructions given by swim teachers and helpful instructions reported by participants considered “active” floaters during floats 3 and 4 in still fresh water (n = 9). Floating competence (assessed using the Moran scale) for float 2 (prior to instruction) and floats 3 and 4 are given ↑ indicates an improvement in floating competency; = indicates no change. Instructions given by the swim teacher and reported by the participant are shaded. Instructions are colour coded as follows: head position; relax; breathing; limb movement; leg position; arm position; practice; core; none.

| **Active** | **Float 2** | **Float Coaching Instructions** | | | | | | **Float 3: Useful instructions** | | | **Float 3** | | **Float 4: Useful instructions** | | | | | **Float 4** | |
| --- | --- | --- | --- | --- | --- | --- | --- | --- | --- | --- | --- | --- | --- | --- | --- | --- | --- | --- | --- |
| P1 | 0 | jawline in the water | Hands sideways so palms across the top of the water, not down and up; aeroplane wings | | tuck your tailbone under, get the tummy up a bit | | legs as wide you can, relax the lower legs | arm & leg movement |  |  | 0 | = | moving from hips, looser arms | | | |  | 2 | ­↑ |
| P3 | 1 | breathe slightly slower | ears going in | Slow down arm action, stroking dogs very gently | spread your legs just slightly | Relax your stomach, relax your core | relax | head back | stroking the dog, kicking a little |  | 3 | ­↑ | relax | | focus on breathing deeply and slowly, | | head back, | 4 | ­↑ |
| P6 | 4 | ears under the water so they are completely filled up with water | | breathe normally | try not to tense your stomach muscles. | hands going sideways, imagine stroking two cats | | putting head back | relaxing | hands in sideways motion not up & down | 4 | = | relax, | | head in water instead of leaning forward | | | 5 | ­↑ |
| P9 | 6 | knees lock a little bit more & let your shoes float up | keep more of your head in the water without arching your back | | relax a little bit more don't feel you've got to move all the time | try and lengthen the spine a little bit more so tuck your tailbone forwards | | bringing legs apart more like breaststroke than front crawl (prob didn't make that much difference) | |  | 6 | = | letting legs hang, but went into a ball so leave at least 90° | | | |  | 6 | ↑ |
| P11 | 6 | head back, fill your ears up with water completely | smaller, gentler, breathing | relax your neck muscles | scull sideways as though you've got 2 cats beside you instead of patting, sideways like aeroplane wings | | let your legs go where they want to go | relax | let body do what it wants to do - let legs drop. | stroke cat - not up and down | 6 | = | stroke cat lower in water | | keeping head right back. | |  | 8 | ­↑ |
| P15 | 3 | try and lengthen the legs | scull a little bit deeper, a little more towards the hips, slow it down | | keep your breathing really relaxed | | relax the neck muscles | sculling deeper, kicking legs | relax more |  | 3 | = | deeper sculling, | relax, | | straight legs | | 4 | ­↑ |

| **Active** | **Float 2** | **Float Coaching Instructions** | | | | | | **Float 3: Useful instructions** | | | **Float 3** | | **Float 4: Useful instructions** | | | **Float 4** | |
| --- | --- | --- | --- | --- | --- | --- | --- | --- | --- | --- | --- | --- | --- | --- | --- | --- | --- |
| P16 | 6 | shallower breath, just control your breathing | relax your neck | Slightly sculling, stroke the cats sideways along their backs, slightly deeper | | lift thighs up and down rather than pushing | | arms further down in water, | shallow vs deep breathing | kick from hips & keep legs straight | 6 | = | hands & legs motion | straighter leg, | regulating breathing | 7 | ­↑ |
| P22 | 6 | lean back | lift your hips very slightly | Breathing nice and relaxed | relax | gentle movements, as little as possible | | expanding chest | breathing deeper | not worrying about legs | 6 | = | lean back | arch back, hips up |  | 6 | = |
| P23 | 3 | head back, ears and hair under | relax your neck muscles | hands flat & sideways stroking 2 cats by your side | Relax your breathing | let your legs come up | push those hips a little bit. | relax neck, | submerge ears | arms side to side (felt effortless) | 5 | ­↑ | relax the neck, | let head go back ears submerge |  | 5 | = |

**Table A1.2.** Floating instructions given by swim teachers and helpful instructions reported by participants considered “passive” floaters during floats 3 and 4 in still fresh water (n = 13). Floating competence (assessed using the Moran scale) for float 2 (prior to instruction) and floats 3 and 4 are given ↑ indicates an improvement in floating competency; = indicates no change. Instructions given by the swim teacher and reported by the participant are shaded. Instructions are colour coded as follows: head position; relax; breathing; limb movement; leg position; hand position; practice; core; none.

| **Passive** | **Float 2** | **Float Coaching Instructions** | | | | | | **Float 3: Useful instructions** | | | | **Float 3** | | **Float 4: Useful instructions** | | | **Float 4** | |
| --- | --- | --- | --- | --- | --- | --- | --- | --- | --- | --- | --- | --- | --- | --- | --- | --- | --- | --- |
| PP2 | 7 | very gentle sideways movement, scull | | Relax | head back |  |  | head back | relax |  |  | 10 | ↑ | relax |  |  | 10 | = |
| P2 | 10 | relax your breathing. | Relax | Head back, ears in the water |  |  |  | being told what to do with head so more confident putting ears in water | | breathing properly | relaxing | 10 | = | being told what to do with head so more confident putting ears in water | breathing properly | relaxing | 10 | = |
| P4 | 6 | head back, ears in water, look up a little bit | relax | breathe nice and steady | if you need to move hands, keep them in line with the body, pretend there are 2 dogs to stroke | | | keep head back, | relax, | keep breathing |  | 8 | ↑ | keep breathing, | keep bead back, | not worrying if legs sink | 8 | = |
| P5 | 7 | It doesn't have to be a star shape, it's whatever feels comfortable to you | | If you need to move your hands, don't too much, stay in line with the body | | it doesn't matter if your legs drop | | none |  |  |  | 9 | ↑ | none |  |  | 8 | ↓ |
| P7 | 6 | lean back a bit further. Look up | Relax | breathing nice and slowly | lying in the sand & you're just pushing bits of sand with your hands to try & keep it in line with the body | | | tilt head back more | sideways arm movement | control breathing | don't try mimic position in video, don't worry about legs if they drop, keeping hips up and level, | 7 | ↑ | tilt head back | move arms sideways, | having practice | 7 | = |

| **Passive** | **Float 2** | **Float Coaching Instructions** | | | | | | **Float 3: Useful instructions** | | | | **Float 3** | | | **Float 4: Useful instructions** | | | **Float 4** | |
| --- | --- | --- | --- | --- | --- | --- | --- | --- | --- | --- | --- | --- | --- | --- | --- | --- | --- | --- | --- |
| P8 | 4 | head back, look up, ears in | Relax | sculling action with your hands, in line with the body, lying in the sand & just sculling your hands in the sand | | | Slow breathing | tilt head back | slower hand movement |  |  | 5 | ↑ | | slowing hands, | letting feet drop, |  | 5 | = |
|  |  |  |  |  | | |  |  |  |  |  |  |  | |  |  |  |  |  |
| P10 | 8 | head back, water is cupping your face, ears will be under | | breathe normally | relax the neck back and relax all the shoulders | |  | head back further | breathe more |  |  | 9 | | ↑ | head back |  |  | 9 | = |
| P12 | 9 | relax your neck muscles. | | breathe normally | |  |  | na |  |  |  | na | |  | relax | breathe |  | 10 | ↑ |
| P13 | 10 | palms down | legs further apart | relax core and neck muscles | | let head go back |  | tried to be more relaxed, relaxed core | | palms down |  | 10 | | = | calm breathing as quickly as possible | relax | palms down not as stable as relaxed position (thumbs up) | 10 | = |
| P14 | 10 | legs a little bit farther apart | try it with your hands behind your head | focus on nice, shallow breathing | keep head right back, hair line |  |  | put hands behind head | relaxed neck | OK to float in a different position from starfish. Legs wider to give more stability | Focus on breathing especially when shivering | 10 | | = | hands behind neck | relaxing, | legs wide | 10 | = |
| P17 | 10 | not as deep breaths, more regulated | | relax your neck muscles and elbows | | push hands down a little bit, keep them nice & flat | | relax neck more, relax wrist and shoulder | | palms down, |  | 10 | | = | relax neck more, relax wrist and shoulder | | palms down, | 10 | = |

| **Passive** | **Float 2** | **Float Coaching Instructions** | | | | | | **Float 3: Useful instructions** | | | | **Float 3** | | **Float 4: Useful instructions** | | | **Float 4** | |
| --- | --- | --- | --- | --- | --- | --- | --- | --- | --- | --- | --- | --- | --- | --- | --- | --- | --- | --- |
| P18 | 8 | small movement like stroking 2 little dogs | put your hip back more, to lift your chest up | find the natural position for you | lean back, think about looking up | relax | relax breathing. | head back more | more relaxed breathing, | trying not to move limbs as much | | 9 | ↑ | head right back | relax | control breathing | 10 | ↑ |
| P24 | 8 | regular, smaller breaths | relax your neck muscles | stroke the cats at your side, do as little as possible | | let your legs go down if that's the way they want to go | | not worry about legs, | not breath holding, regular breathing | |  | 8 | = | relaxing |  |  | 10 | ↑ |
