# Supplementary material for: Inexperienced water users can “Float to Live” in realistic open water conditions
Source: BMC Public Health. 2024 Jul 29;24:2030. doi: 10.1186/s12889-024-19409-6 (PMC11285178; doi:10.1186/s12889-024-19409-6)
Supplement: Supplementary file 2 — Supplementary Material 2. [file 12889_2024_19409_MOESM2_ESM.docx]

**Inexperienced water users can “Float to Live” in realistic open water conditions**

Clare Eglin, Heather Massey, Geoff Long, Adrian Mayhew and Michael Tipton

**Annex 2: Responses of the three participants who undertook four still sea floats (Study 2)**

Three participants undertook all four floats (naïve, following RNLI instruction, following float coaching from a swim instructor and a simulated fall) in still sea water. One participant was very consistent with their perception of floating difficulty and confidence before and after their floats (blue line, Figure A2.1). The other two participants generally reported a decrease in perceived difficulty and an increase in confidence across the first 3 floats. The simulated fall was anticipated to be more difficult, but both participants reported it being easier than they thought.

One participant (green line, Figure A2.1C) showed a slight decline in their float competence following coaching as assessed using the Moran score as they moved their arms more, however they found this helpful (“readjusting with hands and legs to keep in motion with water - balancing, leaning back”) and reduced their perceived floating difficulty. Floating competence was improved in one participant (Orange line, Figure A2.1C), especially after the RNLI video, which they attributed to “controlling their breathing more”. The third participant (Blue line, Figure A2.1C), showed the greatest improvement on their final float with the advice they found helpful being “hand over face when jump in, head back”. The instructions given by the swim teacher and those reported as helpful are shown in Table A2.1. All participants took on board at least one of the recommendations given during the coaching session.

**Figure A2.1.** Individual responses of the three participants who undertook four floats in still sea water

**Table A2.1.** Floating instructions given by swim teachers and helpful instructions reported by participants during floats 3 and 4 in still sea water (n=3). Floating competence (assessed using the Moran scale) for float 2 (prior to instruction) and floats 3 and 4 are given ↑ indicates an improvement in floating competency; = indicates no change. Instructions given by the swim teacher and reported by the participant are shaded. Instructions are colour coded as follows: head position; relax; breathing; limb movement; leg position; hand position; practice; core; none.

| **Sea floats** | **Float 2** | **Float Coaching Instructions** | | |  |  | **Float 3: Useful instructions** | | | **Float 3** | | **Float 4: Useful instructions** | | |  | **Float 4** | |
| --- | --- | --- | --- | --- | --- | --- | --- | --- | --- | --- | --- | --- | --- | --- | --- | --- | --- |
| P19 | 9 | keep hand under the water | | nice slow breaths | relax as much as you can. | | breathing slow and taking breath, | | hands still | 10 | ↑ | breathing slow and taking breath, | | hands still |  | 10 | = |
| P20 | 9 | Head back. | Relax | If you need to manoeuvre yourself from waves, then do so | | | readjusting with hands and legs motion with water | | leaning back | 8 | ↓ | leaning back, | moving arms, | stay calm | breathe | 8 | = |
| P21 | 3 | lie back ears in the water, Look up | get your hips up a little bit more | relax. | gently scull around so that the water's not actually going over your head | relax your breathing | head back, | hips up |  | 4 | ↑ | hand over face when jump in, | head back |  |  | 8 | ↑ |
