# Supplementary material for: Inexperienced water users can “Float to Live” in realistic open water conditions
Source: BMC Public Health. 2024 Jul 29;24:2030. doi: 10.1186/s12889-024-19409-6 (PMC11285178; doi:10.1186/s12889-024-19409-6)
Supplement: Supplementary file 3 — Supplementary Material 3. [file 12889_2024_19409_MOESM3_ESM.docx]

**Inexperienced water users can “Float to Live” in realistic open water conditions**

Clare Eglin, Heather Massey, Geoff Long, Adrian Mayhew and Michael Tipton

**Annex 3: Floats in moving water (Study 1)**

**Methods**

Participants undertaking floats in moving water were also required to be comfortable out of their depth in still water and be able to swim more than 25 m. For safety reasons, participants completed floats in still water (as detailed in the main manuscript) prior to undertaking their floats in moving water.

***Moving sea water***

For the moving sea water condition, six participants undertook floats in Langstone harbour, UK when the current was at its fastest during a rising or falling tide and approximately two hours after they had completed their float in still sea water. Participants had either undertaken four floats in still sea water or four floats in still fresh water followed by two floats in still sea water beforehand. For the first moving water float, participants were transported against the current on a megaSUP to the point of entry into the water. They gently entered the water (sliding off the SUP from a seated position) wearing swim wear and neoprene shoes and commenced their float for up to 2 minutes. Four participants undertook a second float wearing dry shorts and T-shirt and stepped off the paddle board into deep water before commencing their float (the other two participants did not undertake the second float, involving immersion of the face, as there were concerns about the water quality). As with the still water, the participants were instructed to guard their mouth and nose with their hand as they entered the water. No additional instructions/coaching were given during the moving sea water floats as water turbulence and aeration were similar in the still and moving sea water. The study team stayed adjacent to the participants throughout their floats for their safety and to allow data collection.

***Moving fresh water***

Five participants conducted floats in moving fresh water at the bottom of the white-water course at Cardiff International White Water Centre (CIWWC), with the flow set to 4 cubic metres per second, simulating a Grade 1 river which is typical of UK inland water. Participants undertook three floats as follows (and on a separate day to their still water float):

1. No instruction (naïve to moving water)
2. With coaching from a Surf Life Saving Great Britain (SLSGB) instructor on techniques to use in moving water
3. Simulating a fall wearing summer clothing (shorts and T-shirt)

Participants were ferried across to the centre of the water course in an inflatable rescue boat which was then tethered to ensure the participant would enter the water in the middle of the flow of water. For their first two floats participants wore swim wear and neoprene shoes, sat on the sponson with their feet facing down stream and slid into the water as gently as possible. On the final float, participants wore summer clothing (dry shorts and T-shirt), sat on the sponson with their feet facing downstream and fell into the water. Participants floated downstream to another rescue boat, the floating route and duration varied with each individual and during each of their floats, but typically lasted less than one minute. Between each float, the participants immediately dried off, donned a robe and stood in the sun to keep warm.

Additional instructions were given to the participants in the moving fresh water as there was increased turbulence, aeration and obstacles in the moving water compared to the still water. After the first float, an experienced, qualified SLSGB instructor coached participants when out of water on the correct technique to adopt in moving water (“defensive” floating). Although standardised instructions and demonstrations were given, participants were allowed time to ask questions and clarify the actions required. The following instructions were given:

- feet downstream
- chin on chest and look where you are going
- legs together, knees bent and heels lower than bottom
- arms out for stabilisation
- adopt “normal” float as soon as possible when in calmer water

The order of floats undertaken by the participants for each of the conditions is shown in Figure A3.1A in the main manuscript. Data analyses undertaken is as described in the main manuscript.

**Results**

The physical characteristics of the participants and the environmental conditions during the floats in moving water are shown in Table A3.1.

***Moving sea water***

Prior to their floats, no difference in anticipated floating difficulty or confidence was observed between still or moving sea water conditions (Figures A3.1A and A3.1B). Compared to still sea water, floating in moving sea water was perceived to be more difficult (2.0 [0.6] vs 1.2 [0.4]; Figure A3.1F, P = 0.025) and require greater exertion (1.5 [0.8] vs 0.8 [0.4]; Figure A3.1D, P = 0.046) although there was no difference in floating competence (Figure A3.1C) or efficiency (Figure A3.1E). Floating confidence following the floats was not different between the two conditions (Figure A3.1H).

Instructions that the participants reported helping them to float in moving water were similar to those reported for the still sea water (Table A3.2), however a greater proportion reported having their head back (83 % vs 54 %) and relaxing (50 % vs 38 %) as helpful for the moving water.

**Table A3.1** Mean (standard deviation) participant characteristics and environmental conditions during the floats in moving open water in Study. (W = women)

|  |  | **Moving fresh water** | **Moving sea water** |
| --- | --- | --- | --- |
| Participant characteristics | N | 5 (1 W) | 6 (3 W) |
|  | Age (y) | 41.8 (16.5) | 31.5 (14.4) |
|  | Height (cm) | 178.4 (10.4) | 177.5 (9.9) |
|  | Mass (kg) | 80.0 (16.8) | 80.5 (16.4) |
|  | BMI (kg.m^-2^) | 25.0 (3.8) | 25.3 (3.8) |
|  | Σ skinfolds (mm) | 56.9 (7.9) | 83.3 (12.6) |
|  | Body fat (%) | 23.4 (8.2) | 26.2 (4.6) |
|  | Waist:hip ratio | 0.86 (0.06) | 0.89 (0.05) |
| Environmental conditions | Air temperature (°C) | 14.3 (0.5) | 26.7 (5.4) |
|  | WBGT (°C) | 12.3 (0.8) | 22.7 (2.4) |
|  | Average wind speed (knots) | na | 9.5 (5.9) |
|  | Gust speed (knots) | na | 11.8 (4.5) |
|  | Water temperature 5 cm (°C) | 18.1 (0.0) | 21.9 (1.6) |
|  | Water temperature 50 cm (°C) | 17.9 (0.0) | 21.8 (1.6) |
|  | Water flow (m.s^-1^) | 1.4 -1.7 | 0.4 (0.09) |
|  | Average wave height (cm) | na | 5.0 (1.3) |
|  | Maximum wave height (cm) | na | 8.3 (2.1) |
|  | Water specific gravity | 1.000 | 1.027 (0.001) |

***Moving fresh water***

Prior to the floats in moving fresh water there was no difference in anticipated floating difficulty or confidence (Figure A3.2A and B). On the first immersion in moving fresh water, all participants managed to float with their airway clear of the water and the average float competency was the same as in still water (Moran score during Float 3 in still water = 6.8 [1.6]; Float 1 in moving fresh water = 6.8 [1.6]). All but one participant showed an improvement in their moving water technique (Figure A3.2C) following coaching by a SLS-GB instructor (however, this participant reported the floating difficulty was reduced and confidence was increased following instruction). The breakdown of actions correctly undertaken by the participants during their immersions is given in Table A3.2. Floating difficulty showed a trend towards being reduced (χ^2^ = 5.60, P = 0.061, Figure A3.2E). Rating of perceived exertion (Figure A3.2C) and floating confidence (Figure A3.2G) did not differ between the floats. On entry into the water, all participants either had their face splashed or their head was submerged under the water. Breathing interfered with the ability to float during the first float (4 out of 5 participants) and less so following instruction (1/5) or the simulated fall (2/5). There was a trend towards a significant decrease in breathing interference between the first and second floats (χ^2^ = 6.50, P = 0.039; Z = -1.89, P = 0.059).

The instructions that helped the participants float during their first float in fresh moving water were similar to those in still fresh water and included head back (n = 4), relax (n = 3), leg and arm position. The instructions that the participants reported being helpful on their second and third floats reflected the instructions given by the SLS-GB instructor (Table A3.2).

**Table A3.2.** Number (percentage) of participants that correctly undertook the actions recommended by SLS-GB during their moving fresh water immersions as assessed from video analysis and instructions that the participants found helpful during their floats (n = 5).

|  | **Actions correctly performed** | | | **Helpful instructions** | |
| --- | --- | --- | --- | --- | --- |
|  | Naïve | Coaching | Simulated fall | Coaching | Simulated fall |
| Feet downstream | 2 (40 %) | 4 (80 %) | 4 (80 %) | 2 (40 %) | 4 (80 %) |
| Chin on chest | 2 (40 %) | 4 (80 %) | 4 (80 %) | 3 (60 %) | 1 (20 %) |
| Knees bent | 1 (20 %) | 5 (100 %) | 5 (100 %) | 2 (40 %) | 1 (20 %) |
| Legs together |  | 2 (40 %) | 2 (40 %) |  |  |
| Heels lower than bottom | 2 (40 %) | 3 (60 %) | 4 (80 %) |  |  |
| Arms out for stabilisation | 5 (100 %) | 5 (100 %) | 5 (100 %) | 1 (20 %) |  |
| When calm float | 5 (100 %) | 4 (80 %) | 5 (100 %) | 2 (40 %) | 2 (40 %) |
| Defensive position |  |  |  | 1 (20 %) | 1 (20 %) |
| Relax |  |  |  |  | 2 (40 %) |

**Figure A3.1.** Comparison of floats in still and moving sea water. Individual responses are shown for standard floats (wearing swim wear; n = 6) and following a simulated fall (wearing shorts and T-shirt; n = 4) in still and moving sea water. * P < 0.05 between still and moving standard floats, no statistics were conducted on the floats following a simulated fall owing to the small sample size.

**Figure A3.2**. Individual responses of the participants who undertook floats in moving fresh water (n = 5).
